# Study conical pick cutting performance and fatigue life in breaking rock plate process with numerical simulation

**DOI:** 10.1038/s41598-024-51351-w

**Published:** 2024-01-09

**Authors:** Mingyan Wang

**Affiliations:** College of Mechanical and Electronic Engineering, Shandong University of Science and Technology, Qingdao, 266590 China

**Keywords:** Civil engineering, Mineralogy, Petrology

## Abstract

The fatigue life and cutting performance of conical pick is essential for the roadheader machine in the excavation engineering. In rock breaking process, the rock structural conditions has a certain influence on the rock breaking and rock damage with roadheader breaking rock. Established the simulation model of conical pick cutting jointed rock to investigate the influence of the rock joint, rock bedding and confining pressure on the cutting performance and fatigue life of conical pick and rock damage. And the MATLAB is applied to solve the difficulty of rock damage statistical analysis. The research results indicated that the cutting force decreases with the rock joint length increasing, the cutting force increases first and decreases with the rock bedding angle increasing, however, the cutting force decreases first and then increases with the confining pressure increasing. The rock damage has a great relationship with the rock joint, rock bedding and confining pressure. The rock damage increases first and decreases then with the rock joint increasing and the rock bedding angle increasing. However, the influence of confining pressure on rock damage is contrary. Meanwhile, the rock fracture volume is positively correlated with damage value. The research results indicated that the rock structural parameters have an obvious influence on conical pick cutting performance and rock damage.

## Introduction

There are many scholars have done a lot of research about rock breaking with the finite element method. Jaime et al.^1^ pointed out that rock fragmentation and crack propagation are uncertain. Menezes et al.^2^ used the damage constitutive model to establish the conical pick cutting rock simulation model with the ANSYS/LS-DYNA, and found that the rock crack propagation and rock mass separation are mainly determined by the failure criteria. Fourmeau et al.^3^ studied the influence of different mesh element shapes on the breaking rock, and the specific cutting energy consumption obtained from the experiment and simulation is compared. Zhou et al.^4^ simulated the conical pick cutting rock with LS-DYNA and results indicated that the continuous damage constitutive model can well reflect the process of rock fragmentation.

Li et al.^5^ investigated the force of the conical pick when different cutting rollers cut rocks through the simulation software LS-DYNA, studied the impact of conical pick arrangement on cutting performance, and provided theoretical support for the optimization of the conical pick arrangement of the cutting head. Zhu et al.^6^ applied ABAQUS software to simulate rock fragmentation under different cutting conditions and studied the problem of tool wear failure in the process of conical pick cutting rock. Evan et al.^7–9^ studied the influence factors of rock breaking with a conical pick. Zou et al.^10^ studied the debris separation phenomenon in the process of rock fragmentation based on PFC(2D) and pointed out that the cutting depth and cutting angle have a great impact on the average cutting force.

Li et al.^11^ simulated the confining pressure on fracture pressure of conical pick cutting rock with the discrete element method, and analyzed the rock fracture process, cutting force and specific cutting energy of four kinds of rocks under different confining pressure conditions. Wang et al.^12^ pointed out that the rock breaking performance of conical pick is affected by the confining pressure, and proved that the brittleness under biaxial confining pressure is different from that under uniaxial confining pressure through experiments and regression analysis. Jiang et al.^13^ studied the different water jet of cutting head influence on specific cutting energy, compared impact of the water jet on rock breaking performance and determined the best parameters of the water jet assisted rock breaking to improve the rock breaking efficiency. Zeng et al.^14^ studied the cutting force and rock damage with different cutting parameters and investigated the rock breaking performance and fatigue life of conical pick in the process of conical pick cutting rock^15^.

Many scholars have done much studies on rock cutting with conical pick and analyzed the influence of cutting parameters and rock types on the cutting performance of conical pick. However, there are a few numerical simulations on conical pick cutting joint rock. In this paper, the numerical simulation method is applied to study the rock joint length, rock bedding angle and confining pressure on the cutting performance of conical pick. The rock joint length is defined as 0 mm, 6 mm, 10 mm, 18 mm, 30 mm and 50 mm, the rock bedding angle is defined as 0°, 30°, 60° and 90°, the confining pressure is defined as 3 MPa, 5 MPa, 10 MPa and 15 MP, to investigate the influence of rock joint length, rock bedding angle and confining pressure on cutting performance of conical pick and the rock damage.

## Methods

### The rock material constitutive model

Through the constitutive model tests of different rock materials, the RHT constitutive model is selected as the rock material model in this paper. The RHT material constitutive model, coupling shear force and pressure, which is composed of the polynomial hugoniot curve and the compaction relationship *α* describes the pressure. For the compression model, define a variable with an initial value greater than 0 representing porosity, which represents the density fraction of the material and decreases with the increase of pressure. The reference density can be expressed as $\alpha \left({\text{t}}\right)$. The porosity is shown in Eq. (1).

1 $$\alpha \left({\text{t}}\right)= \text{max} \left(\text{1,min}\left\{{\alpha }_{0}\text{,}{\text{min}}_{{\text{s}}\le {\text{t}}}\left[\text{1} + \left({\alpha }_{0}\text{-1}\right){\left(\frac{{\text{p}}_{\text{comp}}\text{-p(s)}}{{\text{p}}_{\text{comp}}-{\text{p}}_{\text{el}}}\right)}^{\text{N}}\right]\right\}\right)$$

Among that, $\alpha$(*t*) represents the pressure at *t* time, *p*_*el*_ represents the initial pore crushing pressure, *p*_*comp*_ represents the compaction pressure, and *N* represents the pore index. Define the cap pressure, and the current pore crushing pressure equation is as shown in Eq. (2).

2 $$  {\text{p}}_{{\text{c}}}  = {\text{p}}_{{{\text{comp}}}}  - \left( {{\text{p}}_{{{\text{comp}}}}  - {\text{p}}_{{{\text{el}}}} } \right)\left( {\frac{{\alpha  - 1}}{{\alpha _{0}  - 1}}} \right)^{{1/N}}  $$

The residual pressure model is determined by the porous density ρ and the specific internal energy *e*, which is according to the input value, it is determined by *B*_*0*_ (${B}_{0}>0$).

3 $$p \left(\rho \text{,e}\right)\text{=}\frac{1}{\partial }\left\{\begin{array}{cc}\left({\text{B}}_{0}+{\text{B}}_{1}\right)\partial \rho \text{e} + {\text{A}}_{1}\eta +{\text{A}}_{2}{\eta }^{2}+{\text{A}}_{3}{\eta }^{3}& \eta > \text{0} \\ {\text{B}}_{0}\partial \rho \text{e} + {\text{T}}_{1}\eta +{\text{T}}_{2}{\eta }^{2}& \eta < \text{0} \end{array}\right.$$

when *B*_0_ = 0, *p* $\left(\rho \text{,e}\right)\text{=}\Gamma \rho {\text{e}}+\frac{1}{\partial }{\text{p}}_{\text{H}}\left(\eta \right)\left(\text{1} - \frac{1}{{2}}\Gamma \eta \right)$, ${\text{p}}_{\text{H}}\left(\eta \right)\text{=}{\text{A}}_{1}\eta +{\text{A}}_{2}{\eta }^{2}+{\text{A}}_{3}{\eta }^{3}$. and $\eta$ $\left(\rho \right)\text{=}\partial \rho \text{/(}{\partial }_{0}{\rho }_{0}\text{)-1}$, the shear strength ${\text{p}}^{*}\text{=p/}{\text{f}}_{\text{c}}$. Meanwhile, $\gamma ={\varepsilon }_{p}^{*}+\left(1-{\varepsilon }_{p}^{*}\right){F}_{e}{F}_{c}$, the failure surface as Eq. (4).

4 $${\sigma }_{f}^{*}\left({p}^{*},{F}_{r}\right)=\left\{\begin{array}{c}\begin{array}{cc}A{\left[{p}^{*}-\frac{{F}_{r}}{3}+{\left(\frac{A}{{F}_{r}}\right)}^{-1/{\text{n}}}\right]}^{n}& 3{p}^{*}\ge {F}_{r}\\ \frac{{F}_{r}{f}_{s}^{*}}{{Q}_{1}}+3{p}^{*}\left(1-\frac{{f}_{s}^{*}}{{Q}_{1}}\right)& {F}_{r}>3{p}^{*}\ge 0\end{array}\\ \begin{array}{cc}\frac{{F}_{r}{f}_{s}^{*}}{{Q}_{1}}-3{p}^{*}\left(\frac{1}{{Q}_{2}}-\frac{{f}_{s}^{*}}{{Q}_{1}{f}_{t}^{*}}\right)& 0>3{p}^{*}>3{p}_{t}^{*}\\ 0& 3{p}_{t}^{*}>3{p}^{*}\end{array}\end{array}\right.$$

among that, ${p}_{t}^{*}=\frac{{F}_{r}{Q}_{2}{f}_{s}^{*}{f}_{t}^{*}}{3\left({Q}_{1}{f}_{t}^{*}-{Q}_{2}{f}_{s}^{*}\right)}$ is failure crushing pressure, ${F}_{r}$ is the dynamic increasing factor, and ${Q}_{1}={\text{R}}_{3}\left(\pi /{6}\text{,}{0}\right)$, ${\text{Q}}_{2}= \text{Q} \left({\text{p}}^{*}\right)$.

In this expression, the relative tensile strength ${\text{f}}_{\text{t}}^{*}$, shear strength ${\text{f}}_{\text{s}}^{*}$ and compressive strength of concrete ${f}_{c}$ and *Q* value to explain the correlation between tensile and shear meridians. For further details, describe the shear and tensile meridian strength reduction factors, such as Eq. (5).

5 $$\text{R}_{3}\left(\theta \text{,p}^{*}\right)\text{=}\frac{{2}\left(\text{1} - {\text{Q}}^{2}\right)\text{cos}\theta +\left({2}\text{Q}\text{-1}\right)\sqrt{{4}\left(\text{1} - {\text{Q}}^{2}\right){\text{cos}}^{2}\theta + \text{5Q}^{2}\text{-4}{\text{Q}}}}{{4}\left(\text{1} - {\text{Q}}^{2}\right){\text{cos}}^{2}\theta +{\left(\text{1-2Q}\right)}^{2}}$$

Among that, the influence of deviator stress tensor *s* on lode angle $\theta$ described as Eq. (6).

6 $$\text{cos3}\theta \text{=}\frac{\text{27det(s)}}{2\tilde{\sigma }{\text{(s)}}^3}, \tilde{\sigma }\left(\text{s}\right)\text{=}\sqrt{3/2\text{s:s}}$$

The relative pressure function reduces maximum strength, $\text{Q=Q}\text{(}{\text{p}}^{*}\text{)}\text{=}{\text{Q}}_{0}+ \text{B} {\text{p}}^{*}$, the relative strain rate coefficient is shown in Eqs. (7) and (8).

7 $$ {\text{F}}_{{\text{r}}} \left( {\dot{\varepsilon }_{{\text{p}}} ,{\text{p}}^{*} } \right) = \left\{ {\begin{array}{*{20}c}    {{\text{F}}_{{\text{r}}}^{{\text{c}}} } & {3{\text{p}}^{*}  \ge {\text{F}}_{{\text{r}}}^{{\text{c}}} }  \\    {{\text{F}}_{{\text{r}}}^{{\text{c}}}  - \frac{{3{\text{p}}^{*}  - {\text{F}}_{{\text{r}}}^{{\text{c}}} }}{{{\text{F}}_{{\text{r}}}^{{\text{c}}}  + {\text{F}}_{{\text{r}}}^{{\text{t}}} {\text{f}}_{t}^{*} }}\left( {{\text{F}}_{{\text{r}}}^{{\text{t}}}  - {\text{F}}_{{\text{r}}}^{{\text{c}}} } \right)} & {{\text{F}}_{{\text{r}}}^{{\text{c}}}  > 3{\text{p}}^{*}  \ge {\text{F}}_{{\text{r}}}^{{\text{t}}} {\text{f}}_{{\text{t}}}^{*} }  \\    {{\text{F}}_{{\text{r}}}^{{\text{t}}} } & { - {\text{F}}_{{\text{r}}}^{{\text{t}}} {\text{f}}_{t}^{*}  > 3{\text{p}}^{*} }  \\   \end{array} } \right. $$

8 $$ {\text{F}}_{{\text{r}}}^{c/t} { = }\left\{ {\begin{array}{*{20}c} {\left( {\frac{{\dot{\varepsilon }_{{\text{p}}} }}{{\dot{\varepsilon }_{{0}}^{c/t} }}} \right)^{{\beta_{c/t} }} } & {\dot{\varepsilon }_{{\text{p}}}^{c/t} > \dot{\varepsilon }_{{\text{p}}} } \\ {\gamma_{c/t} \sqrt {\dot{\varepsilon }_{p} } } & {\dot{\varepsilon }_{{\text{p}}} > \dot{\varepsilon }_{{\text{p}}}^{c/t} } \\ \end{array} } \right.. $$

The parameters involved in the expression are shown in Eq. (9).

9 $${\beta }_{\text{c}}\text{=4/(20+3}{\text{f}}_{\text{c}}\text{)}, {\beta }_{\text{t}}\text{=2/(20+}{\text{f}}_{\text{c}}\text{)}$$

Select the rate parameter by input according to the continuity requirements. The plastic strength factor is shown in Eq. (10).

10 $${\text{F}}_{\text{e}}\left({\text{p}}^{*}\right)\text{=}\left\{\begin{array}{cc}{\text{g}}_{\text{c}}^{*}& {3}{\text{p}}^{*}\ge {\text{F}}_{\text{r}}^{\text{c}}{{\text{g}}}_{\text{c}}^{*}\\ {\text{g}}_{\text{c}}^{*}-\frac{{3}{\text{p}}^{*}-{\text{F}}_{\text{r}}^{\text{c}}{{\text{g}}}_{\text{c}}^{*}}{{\text{F}}_{\text{r}}^{\text{c}}{{\text{g}}}_{\text{c}}^{*}+{\text{F}}_{\text{r}}^{\text{t}}{{\text{g}}}_{\text{t}}^{*}{\text{f}}_{\text{t}}^{*}}\left({\text{g}}_{\text{t}}^{*}-{\text{g}}_{\text{c}}^{*}\right)& {\text{F}}_{\text{r}}^{\text{c}}{{\text{g}}}_{\text{c}}^{*}>{3}{\text{p}}^{*}\ge -{\text{F}}_{\text{r}}^{\text{t}}{{\text{g}}}_{\text{t}}^{*}{\text{f}}_{\text{t}}^{*}\\ {\text{g}}_{\text{t}}^{*}& -{\text{F}}_{\text{r}}^{\text{t}}{{\text{g}}}_{\text{t}}^{*}{\text{f}}_{\text{t}}^{*}>{3}{\text{p}}^{*}\end{array}\right.$$

among that, ${\text{p}}_{\text{c}}^{*}\text{=}{\text{p}}_{\text{c}}/{\text{f}}_{\text{c}}$, and ${\text{p}}_{\text{u}}^{*}\text{=}{\text{F}}_{\text{r}}^{\text{c}}/{\text{g}}_{\text{c}}^{*}+{\text{G}}^{*}{\varepsilon }_{\text{p}}/{\text{f}}_{\text{c}}$, the hardening behavior is linear with plastic strain.

11 $${\varepsilon }_{\text{p}}^{*}= \text{min} \left(\frac{{\varepsilon }_{\text{P}}}{{\varepsilon }_{\text{p}}^{\text{h}}}\text{,}{1}\right)$$

12 $${\varepsilon }_{\text{p}}^{\text{h}}\text{=}\frac{{\sigma }_{\text{y}}\left({\text{p}}^{*}\text{,s,}{\dot{\varepsilon }}_{\text{p}}\text{,}{\varepsilon }_{\text{p}}^{*}\right)\left(\text{1} - {\text{F}}_{\text{e}}{{\text{F}}}_{\text{c}}\right)}{\gamma {3}{\text{G}}^{*}}$$

13 $${\text{G}}^{*}\text{=}\xi {\text{G}}$$

Among that, *G* is the initial shear model of the material, $\xi$ is the hardening attenuation coefficient of the material model. When the hardening state researches the ultimate strength of the failure surface, the damage further accumulates in the process of plastic strain and inelastic loading. The failure plastic strain is shown in Eq. (14).

14 $${\varepsilon }_{\text{p}}^{\text{f}}\text{=}\left\{\begin{array}{cc}{\text{D}}_{1}{\left[{\text{p}}^{*}-\left(\text{1-D}\right){\text{p}}_{\text{t}}^{*}\right]}^{{\text{D}}_{2}}& {\text{p}}^{*}\ge \left(\text{1-D}\right){\text{p}}_{\text{t}}^{*}+{\left(\frac{{\varepsilon }_{\text{p}}^{\text{m}}}{{\text{D}}_{1}}\right)}^{1/{\text{D}}_{2}}\\ {\varepsilon }_{\text{p}}^{\text{m}}& \left(\text{1-D}\right){\text{p}}_{\text{t}}^{*}+{\left(\frac{{\varepsilon }_{\text{p}}^{\text{m}}}{{\text{D}}_{1}}\right)}^{1/{\text{D}}_{2}}>{\text{p}}^{*}\end{array}\right.$$

The damage parameters accumulated by plastic strain are shown in Eq. (15).

15 $$D\text{=}{\int }_{{\varepsilon }_{\text{p}}^{\text{h}}}^{{\varepsilon }_{\text{p}}}\frac{{\text{d}}{\varepsilon }_{\text{p}}}{{\varepsilon }_{\text{p}}^{\text{f}}}$$

The damage surface is shown in Eq. (16).

16 $${\sigma }_{\text{d}}\left({\text{p}}^{*}\text{,s,}{\dot{\varepsilon }}_{\text{p}}\right)\text{=}\left\{\begin{array}{cc}{\sigma }_{\text{y}}\left({\text{p}}^{*}\text{,s,}{\dot{\varepsilon }}_{\text{p}}\text{,1}\right)\left(\text{1-D}\right)+ \text{D} {\text{f}}_{\text{c}}{\sigma }_{r}^{*}{\text{p}}^{*}& {0}\le {\text{p}}^{*}\\ {\sigma }_{\text{y}}\left({\text{p}}^{*}\text{,s,}{\dot{\varepsilon }}_{\text{p}}\text{,1}\right)\left(\text{1-D-}\frac{{\text{p}}^{*}}{{\text{p}}_{\text{t}}^{*}}\right)& \left(\text{1-D}\right){\text{p}}_{\text{t}}^{*}\le {\text{p}}^{*}< \text{0} \end{array}\right.$$

Among that, ${\sigma }_{\text{r}}^{*}\left({\text{p}}^{*}\right)\text{=}{\text{A}}_{\text{f}}{\left({\text{p}}^{*}\right)}^{{\text{n}}_{\text{f}}}$, plastic flow along the direction of deviator stress $\dot{\varepsilon }_{{\text{p}}} \sim {\text{s}}$. However, for tension, the parameter *PFC* can be selected to correspond to the influence value of plastic volumetric strain $\lambda \le 1$ represents the variable, and then the special value $\lambda =1$, $\dot{\varepsilon }_{{\text{p}}} \sim {\text{s}} - {\text{pI}}$.

### The rock material constitutive model

Established the rock damage image statistical software based on MATLAB to solve the rock damage statistical analysis carried out on the rock damage nephogram obtained by the LS-PrePost software. Analyzing the damage range in the rock damage nephogram to calculate the rock damage volume.

**Figure 1:**
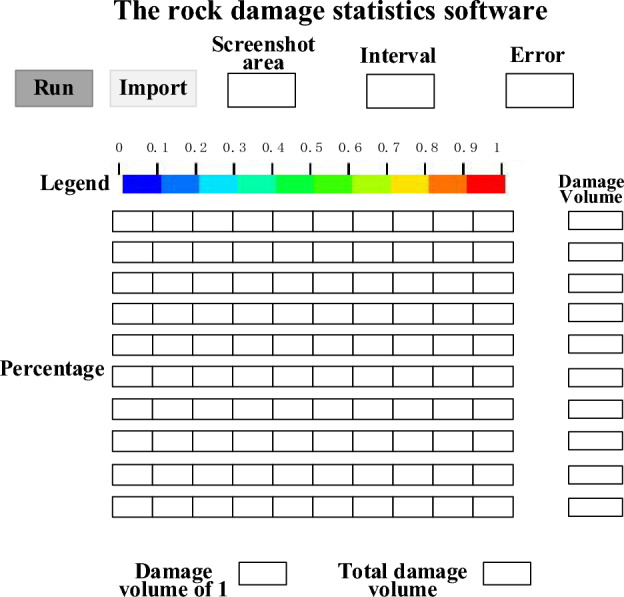
The interface of rock damage statistics software.

Apply the MATLAB function IMread to read the continuously intercepted rock damage cloud image and use them for the loop statement to automatically read the image data in a batch. The interface of rock damage statistics software is shown in Fig. 1. The interface mainly consists of four parts, import section area, interception interval, percentage of each color in the legend, error setting, total damage volume and volume with damage value of 1. The software identifies the area of the imported damage section, solves the damage volume according to the preset damage formula and the necessary input parameters, and outputs it on the interface.

## Established and verified simulation model

### Established numerical simulation

**Figure 2:**
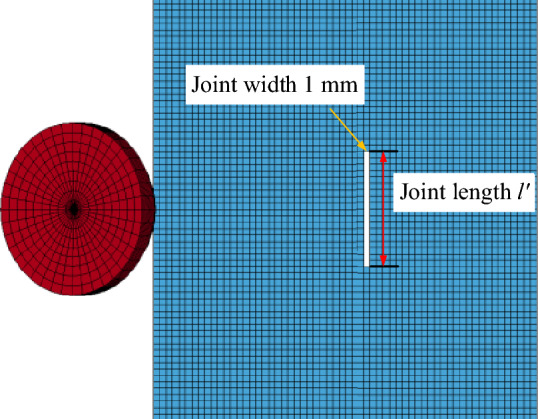
The numerical simulation of conical pick cutting jointed rock.

The numerical simulation of the conical pick cutting rock based ANSYS/LS-DYNA is established to investigate the rock property parameter influence on conical pick cutting performance and rock damage. The conical pick with a tip angle of 80°, cutting angle of 55°, cutting depth of 5 mm, and cutting speed of 3 m/s. And the rock with a length of 60 mm and a width of 100 mm. Therefore, the rock joints, superimposition and confining pressure will be studied. The geometric model of conical pick-rock is imported into the ANSYS/LS-DYNA, and the model is meshed by the Mesh_Tool with Element_Type_164. The material of the conical pick is defined as MAT_RIGID and the rock material is defined as MAT_RHT, and the key parameters is shown as Table 1. The constraints and the no-boundary-reflect-condition are added to the rock, to realize the simulation of natural rock mass. The motion load is added on the conical pick, the rectilinear motion is loaded to conical pick. After the parameters such as time step and calculation time are set according to the numerical simulation, the k file is generated and imported in the ANSYS/SLOVER for the solution. The numerical simulation model of conical pick cutting jointed rock is shown in Fig. 2.

**Table 1:** The key parameters of rock.

Parameters	Density (kg/m^3^)	Tensile strength (MPa)	Shear strength (MPa)	Compressive strength (MPa)	Elastic modulus (GPa)	Poisson’s ratio
Value	2700	6.6	13.7	114	17	0.23

### Verified and modification

Based on the rock cutting bench of conical pick cutting rock, the experiments and numerical simulation of conical pick cutting rock is carried out to verify and modify the numerical simulation model of conical pick cutting rock, which could prove the feasibility of the numerical simulation. The conical pick cutting rock with cutting speed of 3 m/s, cutting depth of 5 mm, cutting angle of 55° and other material parameters are brought into correspondence in the experiment and numerical simulation model.

**Figure 3:**
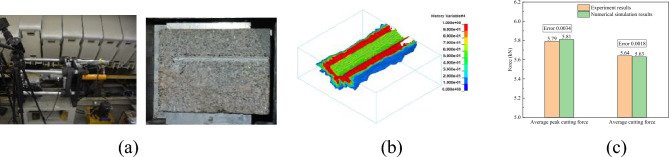
Verification of numerical simulation model of conical pick cutting rock.

The conical pick cutting rock experiment bench and the results of the experiment and numerical simulation are shown in Fig. 3. Compared to the results of the experiments and numerical simulation with the same cutting parameters and material parameters, the cutting groove formed by cutting rock with a conical pick is the same. And the average peak cutting force and average cutting force of conical pick in the cutting rock process are also basically the same, with the error of 0.0034 and 0.0018 respectively less than 0.05, that is, the numerical simulation model of conical pick cutting rock is accurate, as shown in Fig. 3c.

## Results and discussion

The influence of rock structural parameters on conical pick cutting performance and rock damage has been researched. The rock joint length, rock bedding angle and confining pressure have been studied.

### The joint influence on conical pick breaking rock

The conical pick cutting joint rock numerical simulation model is established, with a cutting depth of 5 mm, cutting speed of 3 m/s, the rock joint length of 0 mm, 6 mm, 12 mm, 18 mm, 30 mm and 50 mm to study the rock joint length on conical pick cutting performance and rock damage.

#### The joint influence on cutting force

**Figure 4:**
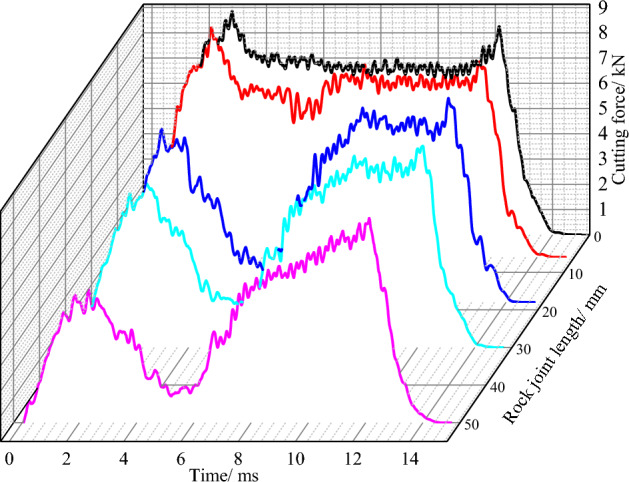
Cutting force curves under different joint lengths.

The cutting force of conical pick cutting rock with different joint length is shown in Fig. 4. Compared the cutting force of conical pick with different rock joint lengths, the rock joint has great influence on the fluctuation and value of cutting force. When there is no joint, the fluctuation of conical pick cutting force is small and the cutting process is relatively stable, owing to that the rock is homogeneous and ideal. However, when the joint length increases, the force curve fluctuation increases. When the cutting time is 4.5 ~ 5 ms, the force curve of the conical pick decreases gradually, which is because of the conical pick cutting joint. Due to the existence of joints, the rock strength is greatly reduced, resulting in a significant reduction in the force of the pick. And with the increase of joint length, the time interval less than a certain cutting force of pick gradually increases. With the increase of joint length, the non-contact part of the rock on both sides of the joint gradually increases, resulting in the change of rock structure and the reduction of rock strength.

**Figure 5:**
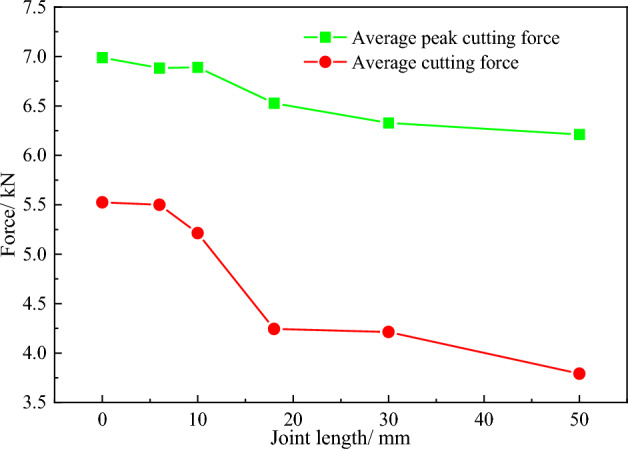
The variation curves of conical pick cutting force with different joint length.

To accurately study the influence of the rock joint length on the cutting force of a conical pick, the statistical analysis of the cutting force of the pick cutting rock with different joint lengths is carried out. The average peak cutting force and average cutting force of the conical pick are applied as important indicators. The average peak force and average force curves with different joint lengths are shown in Fig. 5. The average peak cutting force decreases with the joint length increasing, however, the decreasing range is getting smaller, which indicates that the influence of the increase of joint length on average peak cutting force is gradually reduced, when the joint length increases from 6 to 18 mm. The average force decreases sharply, and with the continuous increase of the joint length, the decreasing range begins to decrease, which is consistent with the fluctuation of the conical pick curve in Fig. 5. When the joint length is less than 6 mm, the force curves fluctuate slightly. With the increase of the joint length, the fluctuation of the conical pick cutting force increases. When the cutting time is 3 ms, the conical pick starts to drop sharply, resulting in a larger fluctuation. When the conical pick does not cut the joint rock, the fluctuation of the conical pick cutting force starts to decrease, and the average force reduction range also starts to decrease. It can be seen that the joint length has a great impact on the peak cutting force in the rock cut by the pick. With the increase of the joint length, the peak cutting force gradually decreases. Therefore, when cutting rocks with different joint lengths, the cutting speed can be appropriately changed to improve the rock breaking efficiency.

#### The jointed rock influence on rock damage

**Figure 6:**
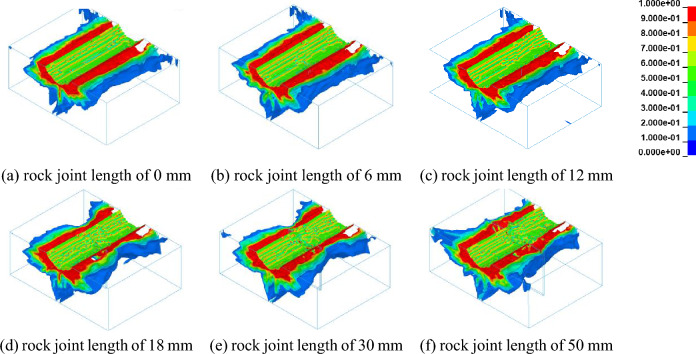
Damage nephogram of rock under different joint lengths.

The numerical simulation is carried out for different joint lengths to quantitatively analyze the influence of joint length on rock damage. Running the ANSYS/LS-DYNA numerical simulation software to obtain the rock damage nephogram with different joint lengths, the rock damage nephogram of the numerical simulation result in the Isosurface_model model is shown as Fig. 6. It can be obtained that the rock damage range is different, and the rock damage propagation law near the joint is different from the damage figure.

**Figure 7:**
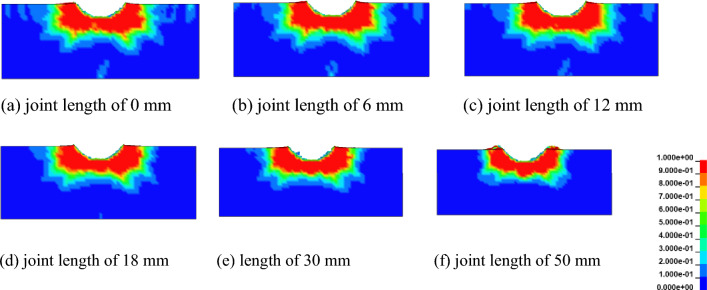
Damage nephogram of rock cross section under different joint lengths.

To study the influence of joint length on rock damage propagation, the rock damage cross-section distribution with different joint lengths is shown in the Fig. 7. It can be concluded that the rock damage area is located around the broken area of the pick, and the rock damage volume with the damage value of 1 occupies the main position, and the rock damage range is similar to the oval extending outward. With the increase of joint length, the damage range of rock tends to decrease. Owing to the increase in joint length changes the rock properties, and the rock strength decreases, the damage propagation along the cutting direction (negative direction of the X axis) is accelerated when the rock is cut by the pick. However, along the Y-axis direction, the rock damage range on both sides of the pick tends to decrease.

**Figure 8:**
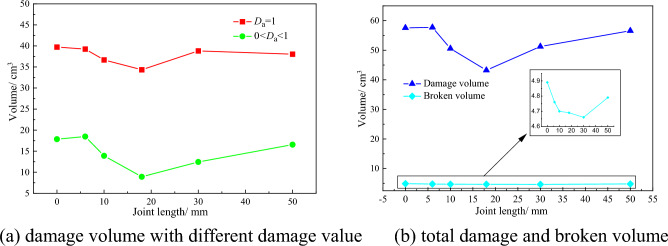
Curves of rock damage and broken volume under different joint lengths.

In order to study the damage volume of rock under different joint lengths, and quantitatively analyze the influence of joint length on the damage volume of rock. According to the statistical method of rock damage, set the interception interval to 1 mm, intercept the damage nephogram obtained after the completion of numerical simulation, and obtain the required number of damage nephogram cross sections. Import the obtained section nephogram into the rock damage statistics software, input the necessary parameter section area *S* = 3000 mm^2^, intercept the interval *d* = 1 mm, and get the rock damage volume under different joint lengths. The number of failed units can be obtained by viewing the Message file in the post-processing software LS-PrePost, which is used for the statistics of rock breaking volume. The statistics of rock damage volume and rock fracture volume under different joint lengths are shown in Fig. 8.

The numerical simulation results draw the relationship between the rock damage volume and fracture volume and joint length, as shown in Fig. 8. It can be seen from Fig. 8a that the damage volume of rock with damage value of 1 still dominates. With the increase of joint length, the proportion of damage volume with damage value of 1 decreases. It can be concluded that the joint length has a great impact on the total volume of rock damage from Fig. 8b. With the increase of the joint length, the rock damage volume shows a trend of decreasing first and then increasing, but the increase gradually slows down. Because when the joint length is small, the impact on the physical properties of the rock is small. With the increase of the joint length, the impact gradually increases. The existence of joints hinders the expansion of rock damage, leading to the reduction of damage volume. When the joint length is larger than the rock breaking range, the joint will no longer hinder the expansion of rock damage along the X-axis direction, and the rock damage volume will gradually increase, but the increasing trend will be smaller and smaller. The fracture volume in Fig. 8b decreases first and then increases with the increase of joint length. In general, the impact of joint length on rock fracture volume is limited.

According to the results of numerical simulation about conical pick cutting joint rock with different joint length, it can be concluded that the joints have great influence on rock damage. With the increase in joint length, the average peak cutting force decreases, and the rock damage volume decreases.

### The bedding influence on conical pick breaking rock

**Figure 9:**
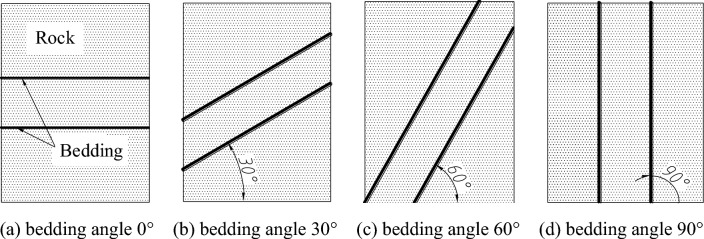
Diagram of rock bedding angle.

There are different angles between the rock bedding and the horizontal plane in the rock, therefore, the influence of rock bedding angle on rock breaking should be researched. The bedding angles are defined as the 0°, 30°, 60°, and 90°, shown in Fig. 9.

#### The cutting force with different rock bedding angles

**Figure 10:**
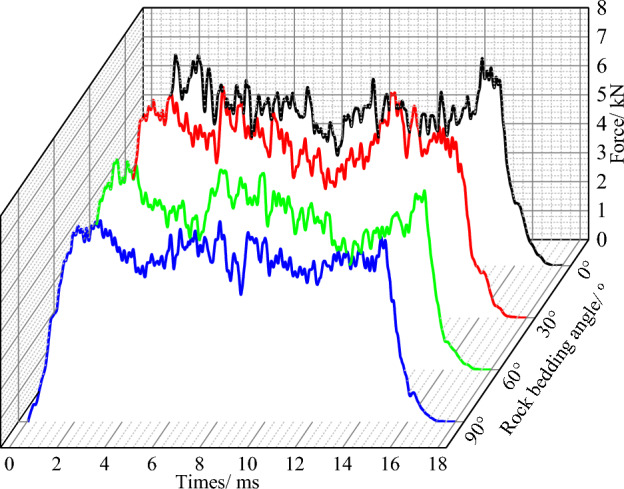
Cutting force curves under different bedding angles.

In the process of conical pick cutting rock with different rock bedding angles, the cutting force cures of the conical pick are shown in Fig. 10. Comparing the cutting force cures with different rock bedding angles, the magnitude and fluctuation of cutting force curves are different. When the rock bedding and cutting direction is between 0° and 90°, the difference between the peak force and trough force of the cutting force is relatively stable, the fluctuation of the conical pick cutting force is small, and the cutting process is relatively stable. With the increase of the bedding angle, the rock strength decreases first and increases then, and the rock is destroyed along the bedding plane. When the bedding angles between 30° and 60°, the fluctuation of the conical pick cutting force angle increases. The change of the cutting force is complex. Although the cutting depth and other parameters of the conical pick have not changed, the force of the pick is greater than the bedding angle is 0 and 90°. The effective area of conical pick cutting rock increases, and the area of the rock peeling section increases, resulting in the increased fluctuation of the conical pick cutting force, and the cutting force of conical cutting increases.

**Figure 11:**
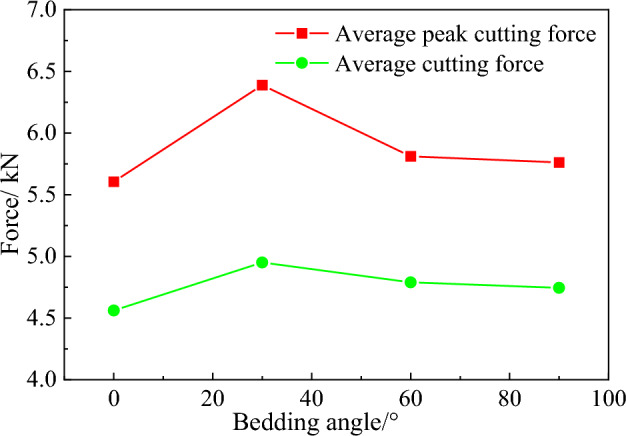
Variation curves of statistical results of average peak cutting force under different bedding angles.

The average peak cutting force and average cutting force curves of conical pick cutting rock with different bedding angles are shown in Fig. 11. When the conical pick cutting rock, the average cutting force and average peak cutting force are greater than the force of the conical pick with the conical pick cutting rock with bedding. It is indicated that the rock bedding reduces the rock strength, which is consistent with the actual rock breaking situation of the conical pick. According to the meaning of the mean square deviation formula, the mean square deviation represents the fluctuation of the cutting force of the pick. The smaller the fluctuation, the smaller the wear of the pick, and the longer the life of the pick. According to the meaning of the mean square deviation formula, the mean square deviation represents the fluctuation of the conical pick cutting force. the smaller the fluctuation, the smaller of conical pick wear and the longer the fatigue life of the conical pick. When the bedding angle is 0°, the mean square deviation of the rock cutting with a conical pick is the smallest, and the wear of the conical pick is the smallest. With the increase of the bedding angle, the average peak force, the average force and the mean square deviation of the rock cutting with a conical pick are increased compared with that of the rock without bedding.

#### The rock damage with different rock bedding angles

**Figure 12:**
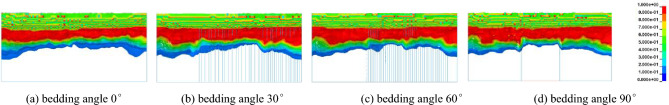
Damage nephogram of rock under different bedding angles.

The rock bedding angle is studied based on numerical simulation with the same cutting parameters to quantitatively analyze the influence of rock bedding on rock damage. Running the numerical simulation software ANSYS/LS-DYNA obtain the rock damage nephogram with different rock bedding angles. In the Isosurface_model display mode, the broken rock damage nephogram is dissected, and the numerical simulation results of rock damage are shown in Fig. 12. It can be obtained that when the conical pick cutting rock with a rock bedding angle of 0°, the rock damage range is relatively uniform and the damage propagation fluctuation is small from Fig. 12a. And compared with Fig. 12b, it is obvious that the rock damage range when the rock cutting rock with a rock bedding angle of 30°, however, the bedding plane has a certain barrier effect on rock damage expansion. With the rock bedding angle increasing to 60°, there is no large fluctuation in the damage propagation of rock at the bedding plane. However, while the rock bedding angle increases to 90°, the rock damage propagation has an obvious boundary, the rock damage propagation at the bedding plane is restricted and the rock damage range is reduced. It can be seen from the rock damage propagation is different, and the rock damage law near the rock bedding is different.

**Figure 13:**
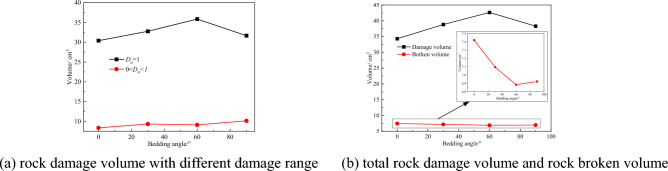
Curves of rock damage and broken volume under different bedding angles.

To study the rock bedding angle influence on rock damage volume, the rock damage quantitatively analyze method is applied to investigate the rock bedding angle influence on rock damage. The damage nephogram obtained after the completion of the numerical simulation is intercepted at an internal of 1 mm with damage nephogram sections. The obtained section nephogram is imported into the rock damage statistics software to obtain the rock damage volume under different rock bedding angles. The number of failure elements can be obtained by viewing the Message file in the LS-PrePost, which is used for the statistics of rock fracture volume. The statistics of rock damage volume and rock fracture volume with different rock bedding angles are shown in Fig. 13.

The relationship between rock damage volume, rock damage, and rock bedding angle of the numerical simulation results in Fig. 13. It can be seen from Fig. 13a that the damage volume of rock with a damage value of 1 occupies the main position. With the increase of bedding angle, the damage volume with damage value of 1 increases first and then decreases, and the damage volume with a damage value of less than 1 gradually increases, but the increase is small. According to Fig. 13b, with the increase of bedding angle, the total volume of rock damage still increases first and then decreases. This is because the existence of a bedding plane changes the physical properties of rock, and the rock strength decreases. Because of the existence of the bedding angle, the effective area of rock cut by the pick increases, resulting in the maximum total volume of rock damage when the bedding angle is 60° with the same cutting speed. The broken volume curve in Fig. 13b is approximately a horizontal line. After zooming, it can be seen that the broken volume first decreases and then increases with the increase of the bedding angle, but the change range is small, and the bedding angle has little impact on the broken volume of rock.

The bedding has a certain influence on the mechanical properties of rock breaking and rock damage of the pick. And the average peak force and average force of the pick when cutting layered rock are less than that of the pick when cutting homogeneous rock. With the increase of bedding angle, the average peak force of the pick first increases and then decreases, and the rock damage volume also shows a trend of first increasing and then decreasing.

### The lateral pressure on conical pick breaking rock

**Figure 14:**
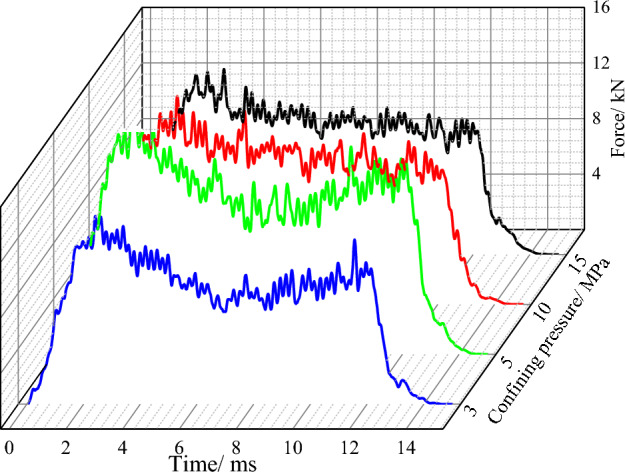
Cutting force curves under different lateral pressure.

The confining pressure is defined as 3 MPa, 5 MPa, 10 MPa and 15 MPa to study the influence of the confining pressure on conical pick cutting performance and rock damage. The cutting force curves with different confining pressure are shown in Fig. 14. Comparing the cutting force curves with different confining pressure curves, the cutting force value and fluctuation are different. In the process of cutting force curve rising stage, the conical pick cuts into rock, and the force of the conical pick increases with the intrusion of the conical pick. At the same time, microcracks and broken areas begin to appear inside the rock and gradually penetrate. By observing the four curves, it can be found that when the confining pressure is small, the fluctuation amplitude of the conical pick cutting force is small. With the confining pressure increasing, the rock strength increases and fluctuation gradually increases.

**Figure 15:**
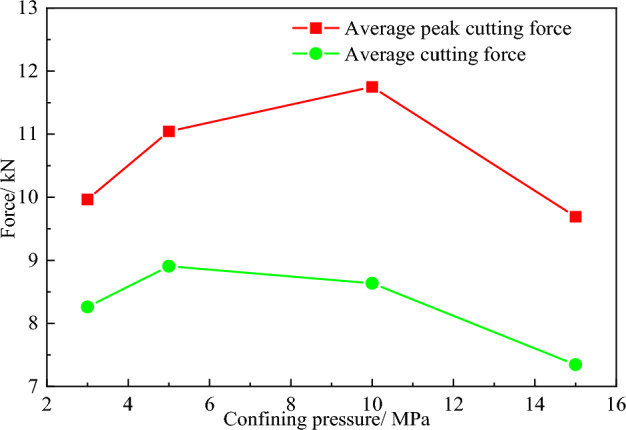
The curves of average peak cutting force, average cutting force and confining pressure.

In order to quantitatively analyze the influence of different confining pressure on the mechanical characteristics of rock cutting with a conical pick, the average peak cutting force and average cutting force of conical pick are obtained by statistics analysis of conical pick cutting rock. The average peak cutting force and average cutting force with different confining pressure are shown in Fig. 15.

In the range of confining pressure studied in the paper, with the increase of confining pressure, the average cutting force and average peak cutting force first increase and then decrease, but the change range of the average force is smaller than the average peak force. With the increase of confining pressure, the difficulty of breaking rock increases and the fluctuation of conical pick cutting force increases.

**Figure 16:**
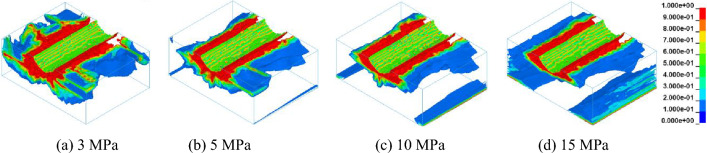
Damage nephogram of rock under different lateral pressure.

The influence of confining pressure on rock damage is quantitatively analyzed by numerical simulation or conical pick cutting rock with different confining pressures. Running the ANSYS/LS-DYNA numerical simulation software to obtain the rock damage nephogram with different confining pressures. In the Isosurface_model display mode, the numerical simulation results of rock damage are shown in Fig. 16. It can be seen that the range of rock damage with different confining pressure from Fig. 16. With the increase of confining pressure, the rock damage propagation change law is changed.

**Figure 17:**
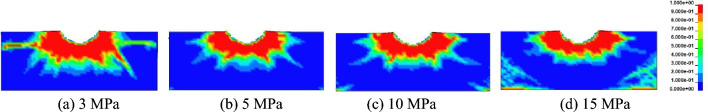
Damage nephogram of rock cross section under different lateral pressures.

In order to study the influence of confining pressure on rock damage propagation, the cross sections distribution of rock damage under different confining pressure is shown in Fig. 17. It can be concluded that the range of rock damage is not only around the broken area of the conical pick, but the rock damage proportion with a damage value of 1 change from Fig. 17. With the increase of confining pressure, the rock damage propagation law changes, and the damage range of rock in cross section decreases first and increases then. Due to the existence of confining pressure, the void and microcracks in the rock are compacted, increasing elastic modulus. With the continuous increase of confining pressure, the rock is significantly deformed, leading to an increase in rock damage.

**Figure 18:**
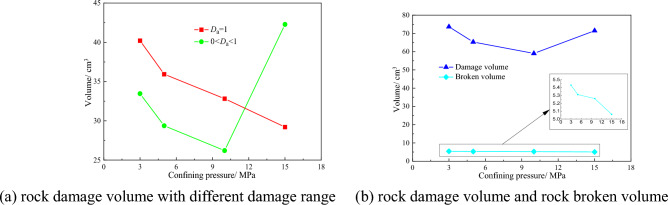
Curves of rock damage and broken volume under different lateral pressure.

To study the rock damage value with different confining pressure and quantitatively nephogram analyze the influence of confining pressure on rock damage. The damage nephogram obtained after the completion of the numerical simulation is intercepted at an interval of 1 mm, and the obtained section nephogram is imported into the rock damage statistics software to obtain the rock damage volume under different confining pressures. The number of failed units can be obtained by viewing the Message file in the post-processing software LS-PrePost, which is used for the statistics of rock breaking volume. The statistics of rock damage volume and rock breaking volume under different confining pressures are shown in Fig. 18.

The relationship between the rock damage volume and confining pressure, rock broken volume and confining pressure is shown in Fig. 18. It can be seen from Fig. 18a that with the increase of confining pressure, the volume of rock with damage value of 1 gradually decreases, and the proportion range has a decreasing trend. The damage volume with damage value less than 1 first decreases and then increases. The existence of confining pressure changes the law of rock damage propagation. It can be seen from Fig. 18b that the size of confining pressure has a great impact on the total volume of rock damage. With the increase of confining pressure, the total volume of rock damage decreases first and then increases. This is because the existence of confining pressure increases the compressive strength and elastic modulus of rock. With the further increase of confining pressure, the rock itself begins to appear damage, thus increasing the damage volume of rock. In Fig. 18b, the broken volume of rock gradually decreases with the increase of confining pressure, but the decrease is small, and the impact of confining pressure on the broken volume of rock is small. The confining pressure has a certain impact on the mechanical characteristics of rock breaking and rock damage of the pick. With the increase of the confining pressure, the average peak force of the pick first increases and then decreases, and the rock damage volume first decreases and then increases.

## Conclusion

The RHT constitutive model is used to establish the finite element numerical model of single pick linear cutting rock. The joint simulation is carried out through the dynamic simulation software ANSYS/LS-DYNA and MATLAB, and the influence of joint length, bedding angle, and confining pressure on the mechanical characteristics of rock cutting and rock damage of the pick is analyzed. The simulation results show that the average peak force of the pick decreases with the increase of the joint length, and the peak cutting force increases first and then decreases with the increase of the confining pressure and the bedding angle; The damage volume of rock decreases first and then increases with the increase of joint length and confining pressure, and increases first and then decreases with the increase of bedding angle; The joint length, bedding angle and confining pressure have little influence on the broken volume of the pick.

## Data Availability

The data used to support the findings of this study are included in the article.
